# Prevalence and Risk Factors for Obesity After Liver Transplantation: A Single-Center Experience

**DOI:** 10.5812/hepatmon.7569

**Published:** 2013-08-01

**Authors:** Mesut Akarsu, Yasin Bakir, Sedat Karademir, Tarkan Unek, Aylin Bacakoglu, Ibrahim Astarcioglu

**Affiliations:** 1 Division of Gastroenterology, Faculty of Medicine, Dokuz Eylül University, Izmir, Turkey; 2 Department of Hepatobiliary Surgery and Liver Transplantation, Dokuz Eylül University, Izmir, Turkey

**Keywords:** Liver Transplantation, Obesity, Immunosuppressive Agents, Metabolic Diseases

## Abstract

**Background:**

The study of weight gain after transplantation and its associated factors is necessary to propose strategies to prevent and treat this problem.

**Objectives:**

This study aims to investigate factors affecting the development of obesity after liver transplantation (LTx).

**Patients and Methods:**

Medical records of 343 liver transplantation cases, which were followed between January 2001 and January 2010 at Dokuz Eylul University, were retrospectively analyzed. Patient pre-liver transplantation height, body weight, body mass index (BMI) measurements, as well as changes in body weight at the beginning, 6 months, 12 months, and 5 years post-transplantation were observed. BMI measurements with records of immunosuppressive therapies were obtained.

**Results:**

The study was carried out with the records of 226 patients. 151 patients (66.8%) were male; 75 (33.2%) were female. The mean age was 46.19 ± 10.2 years. 123 of these liver transplants were performed from living donors, while 103 were from cadaveric donors. The causes of liver transplantation were hepatitis D virus (HDV) infection (28%), hepatitis B virus (HBV) infection (24%), hepatitis C virus (HCV) infection (24%), alcoholic liver disease (9%), cryptogenic liver disease (9%), autoimmune hepatitis (4%), and other (2%). In this study, the prevalence of obesity was 21% at the end of the second year, decreasing to 14% by the end of the fifth year. The mean BMI gradually increased during the follow-ups, reaching 25.1 kg/m² and 26 kg/m² six months after liver transplantation and at the end of the first year, respectively (P < 0.002). Obesity developed in 18.2% of post-transplant patients who were receiving a calcineurin inhibitor (CNI). Regarding the development of obesity after transplantation, no statistically significant difference was found between patients using cyclosporine (CsA) and tacrolimus (TAC) (P = 0.07). Six months after liver transplantation, the mean body weight gain in the groups receiving steroids and not receiving steroids were 4.71 kg and 2.7 kg, respectively (P = 0.03). In the post-transplant period, there was no significant difference in patients who had received TAC and CsA for development of diabetes mellitus (DM), hypertension (HT), or hyperlipidemia (HL) (P = 0.30).

**Conclusions:**

Obesity prevalence before and after liver transplantation was comparable. Education of obese patients prior to surgery and recommendation of medical nutrition therapy should be appropriate. Similar medical care for the non-obese subjects could prevent increase in obesity prevalence. Non-corticosteroid immunosuppressive agents had no significant effect on the development of weight gain and obesity. Avoiding the use of long-term steroid therapy and obesity education are the key measures for preventing obesity after liver transplantation.

## 1. Background

Liver transplantation is the most effective treatment method for acute or chronic end- stage liver disease (1). New health problems may arise in post-transplant patients during follow-ups due to extended lifespan and immunosuppressive treatments (1, 2). Weight gain and obesity are some of the most common health problems of liver transplantation patients (1, 3, 4). In our country, Turkey, new health problems are inevitable in the course of immunosuppressive treatments as a result of successful liver transplantations and longer survival time. Evaluating obesity and other factors affecting obesity (immunosuppressive treatments, diabetes mellitus, and hyperlipidemia) after liver transplantation at Dokuz Eylül University will thereby enable us to take medical precautions and apply dietary programs to decrease the occurrence of obesity. Developments in surgery techniques and increasing experience in pre-operative patient care, along with new immunosuppressive medications, have increased survival rates and also improved post-transplant quality of life, significantly. Today, the survival rates at experienced centers at 1 and 5 years can reach 85–90% and 70–80%, respectively (5).

### 1.1. Weight Gain and Obesity After Liver Transplantation

Obesity is seen in approximately 20% of patients after liver transplantation (6). A considerable amount of weight is gained in the first year following the operation; the weight gain plateaus after the first year (7). Many factors including sedentary lifestyle, improvement in nutrition, and immunosuppressive medications are thought to play a role in obesity (1, 3, 4). It is known that immunosuppressive medications used after liver transplantation have many side effects. However, the effect of different types of immunosuppressive therapies on weight gain has not been clarified yet (8).

## 2. Objectives

The aim of this study was to investigate factors affecting the development of obesity after liver transplantation (LTx).

## 3. Patients and Methods

In this retrospective study, medical records were evaluated for 343 liver transplants (338 patients) that underwent this procedure at the Hepatopancreatobiliary Surgery Unit and were followed up at the Liver Transplantation Outpatient clinic at Dokuz Eylül University between January 2001 and January 2010. The study was approved by the local ethics committee of Dokuz Eylul University Hospital.

### 3.1. Inclusion Criteria

Patients older than 18 years who were monitored for ≥ 1 year after transplantation were included in the study.

### 3.2. Exclusion Criteria

Patients who had multiple organ transplantations (5 patients with retransplantation), patients < 18 years old (17 patients), follow-up period < 1 year (71 patients), and patients with insufficient data (19 patients) were excluded. There were a total of 226 eligible patients. Patient’s age, sex, disease etiology, donor type, pre-operative Child-Pugh score, MELD score, pre-transplant height, weight, and body mass index measurements were collected from their charts. Post-transplant weight and laboratory data including fasting glucose and serum lipid levels were assessed in the 1st, 6th, and 12th months and yearly thereafter for a total of five years. We also noted the medications used, including corticosteroids and other immunosuppressive treatments.

### 3.3. Definitions

Hypertension: Average systolic BP ≥ 140 mmHg or average diastolic BP ≥ 90 mmHg, or receiving treatment for HT.

Dyslipidemia: Total cholesterol level ≥260 mg/dl or low-density lipoprotein (LDL) cholesterol ≥160 mg/dl or the use of lipid-lowering medication.

Diabetes mellitus: Current use of medications prescribed to treat DM or fasting serum glucose levels ≥ 126 mg/dL.

Obesity: Body mass index (BMI) ≥ 30.

### 3.4. Statistical Analysis

The distributions of variables were evaluated by Kolmogorov–Smirnov normality testing and Q–Q plots. Considering sample sizes and variant ranges, Student’s t-test, ANOVA test, Mann–Whitney U test, and Kruskal test were used in group comparisons. A paired samples t-test and Wilcoxon test were used to compare differences between the correlated groups. The Bonferroni procedure was used as a post-hoc test. Cox regression test was used for multivariate analysis. Categorical variables were analyzed by the Chi Square test and Fisher’s exact test. Parametric data, mean ± standard deviation and categorical data were expressed as percentages. Statistical analysis was conducted by SPSS (Statistical Package for the Social Sciences for Windows) version 15.0. Results were considered statistically significant at P < 0.05. 

## 4. Results

### 4.1. Demographic Features of Patients

The study was carried out with the records of 226 patients. 151 patients (66.8%) were men, while 75 (33.2%) were female. The mean patient age was 46.19 ± 10.2 years. 123 of these liver transplantations were performed from living donors; 103 were from cadaveric donors. Child-Pugh scores were child-A in 13 patients (5.8%), child-B in 130 patients (57.5%) and child-C in 83 patients (36.7%). The MELD score mean was 15.67 ± 5.96: 15.42 ± 5.94 for male patients and 16.17 ± 6.01 for female patients.

### 4.2. Etiology of Patients

The causes of liver transplantation were hepatitis D virus (HDV) infection (28%), hepatitis B virus (HBV) infection (24%), Hepatitis C virus (HCV) infection (24%), alcoholic liver disease (9%), cryptogenic liver disease (9%), autoimmune hepatitis (4%), and other (2%).

### 4.3. Patient Body Weight and Body Mass Index Measurements

Mean body weights (BW) before and after transplantation of the patients included in the study (n = 226) and the changes over time are shown in Table 1. The mean weight of the patients at the time of transplantation was 72.1 ± 12.9 kg (male BW: 75.5 kg, female BW: 65.3 kg). 

In the 1st month of control after the operation, we determined a mean BW of 66.4 ± 11.0 kg and average weight change of 5.7 ± 6.2 kg, which were statistically significant (P = 0.00).

Patient mean BMI before transplantation was 25.7 ± 4.2 kg/m² (male BMI: 25.6 kg/m², female BMI: 25.7 kg/m²) (Table 2). Mean BMI within the 1st month after transplantation was found to be 23.7± 3.6 kg/m². The mean BMI gradually increased after liver transplantation, reaching 25.1± 3.8 kg/m² at six months and 26.2 ± 4.0 kg/m² at one year. A statistically significant increase was observed in patient BMI values (P < 0.002). However, BMI measurements in the 2nd–3rd and 4th–5th years after transplantation did not show a statistically significant difference with regards to BMI change (P > 0.05). Before liver transplantation, 13 patients had BMI < 20 kg/m (5.8%), 84 (37.1%) were overweight (BMI ≥ 25 and < 30), and 33 patients had BMI ≥ 30 kg/m². In the first month after transplantation, there were 13 (5.8%) obese patients. This number increased to 29 (12.8%) in the sixth month, 37 (16.4%) in the first year, and 40 (21.0%) in the second year. Increase in the BMI was higher in the first year in comparison to the second year after liver transplantation (P > 0.05). 

**Figure 1.**
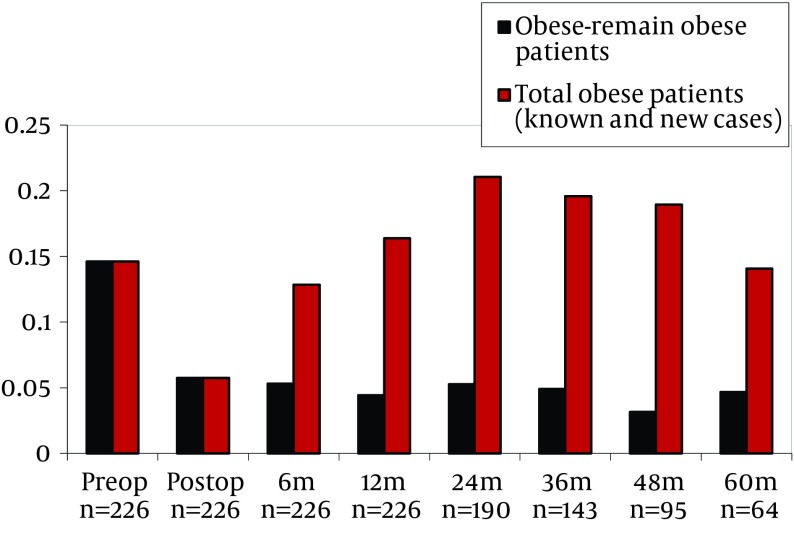
Known and New Obese Cases in Post-Transplantation Follow-Up (Preop: Before Liver Transplantation, Postop: After Liver Transplantation, M: Month)

Data of pre-transplant obese subjects who remained obese after transplantation and all obese subjects (known and new subjects) is shown in Table 3 and Figure 1. Multivariate analysis showed that, age, gender, CsA, TaC and sirolimus predicted obesity. Risk of obesity was higher for elderly and male patients and was lower for subjects used to TAC, CsA and sirolimus (Table 4). In the pre-transplantation obese group, multivariate analysis demonstrated that, age, gender, CsA, TaC and sirolimus significantly predicted obesity. Subjects with pre-transplantation obesity had 4.37 times higher risk of post-transplantation obesity (Table 4). In the multivariate analysis, when pre-transplantation obese group was excluded, it was demonstrated that age, gender, Tac, Cya and sirolimus significantly predicted obesity. In this group of subjects, risk of developing obesity was found to be 2.87 times higher in men than in women (Table 4). 

### 4.4. Immunosuppressive Medication

Immunosuppressive medications used after liver transplantation are primarily corticosteroids as well as mycophenolate mofetil, calcineurin inhibitors (tacrolimus, cyclosporine) and mTOR inhibitors (sirolimus, everolimus). In the first month after liver transplantation, 216 patients (99.5%) used steroids, 199 patients (88.0%) used MMF, 167 patients (73.8%) used TAC, 56 patients (24.7%) used CsA and 10 patients (4.4%) used mTOR inhibitors.

### 4.5. Calcineurin Inhibitors (CNI)

There was no statistically significant difference in mean weight gain between the patients using TAC and CsA as immunosuppressive treatments (P = 0.06). Obesity developed in 18.2% of the patients who used CNI (TAC or CsA) after transplantation and these patients were not obese at the time of the operation (Table 5). Furthermore, obesity developed in 21 of 117 patients (17.9%) who received TAC- based treatment and in 6 of 31 patients (19.4%) who received CsA-based treatment. Concerning the development of obesity after transplantation, there was no statistically significant difference between patients who used tacrolimus and patients who used cyclosporine (P = 0.07). 

### 4.6. Corticosteroid

164 of 226 patients (72.5%) used a steroid only for the first six months, while 49 patients (21.6%) used a steroid for the entire first year. The patients who received a steroid for the first six months were compared to non-users regarding their weight change in the first and the sixth month after transplantation (Table 5). The mean weight gain of the patients who used a steroid for six months was 4.71 ± 5.75 kg, compared to 2.77 ± 6.98 kg for patients in the non-steroid group. The difference between these two patient groups was statistically significant (P = 0.03). At the end of the first year, the mean weight gain for the steroid group was 7.84 ± 7.18 kg compared to 5.43 ± 7.20 kg for the non-steroid group. A significant weight gain difference between these two patient groups was found (P = 0.02). The mean weight gain of the patients who used a steroid for the first year (21.6%) was 7.14 ± 7.84 kg, close to that of patients who did not use a steroid (7.19 ± 7.10 kg). Statistical analysis did not show a significant difference between these two groups (P = 0.59). 

### 4.7. Development of Metabolic Disease

**Figure 2.**
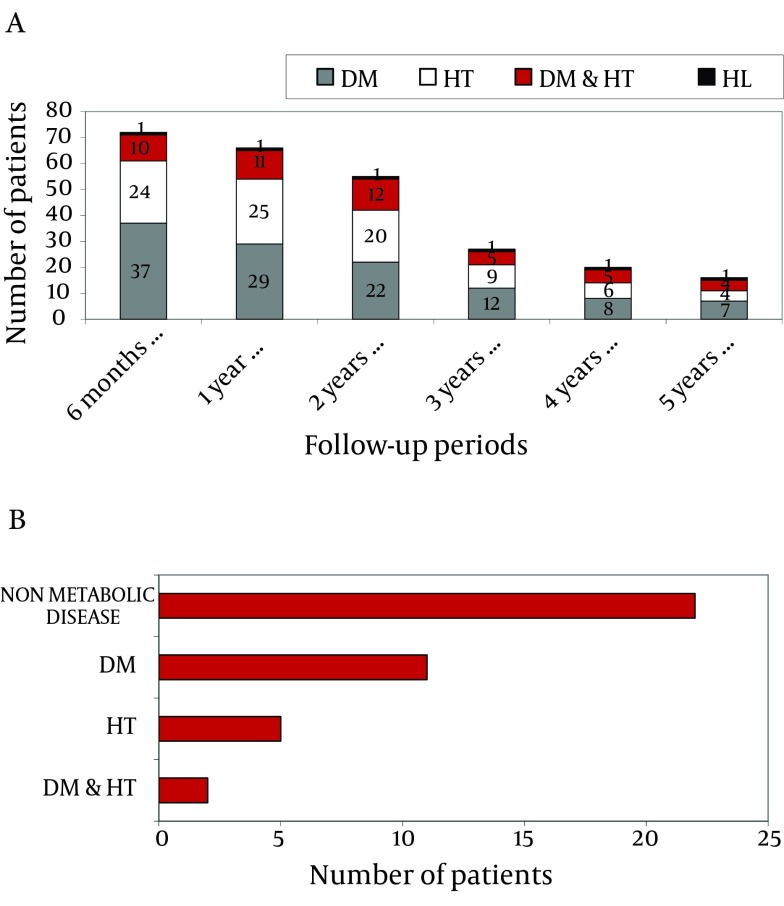
A) Metabolic diseases over the years in post-transplant patients, B) Metabolic diseases after liver transplantation in obesity developed patients

The entire population of the study with 226 patients, included 41 patients (18.1%) with diabetes mellitus, 15 patients (6.6%) with hypertension, and 1 patient with hyperlipidemia before liver transplantation. Patients’ metabolic diseases over the years are shown in Figure 2 a, based on their retrospective data. Within six months of liver transplantation, 38 (16.8%) of the 226 patients monitored had DM (diabetes mellitus), 24 (10.6%) had HT (hypertension), 10 (4.4%) had DM and HT, and one (0.4%) had HL (hyperlipidemia). One year after transplantation, 29 patients (12.8%) had DM, 25 (11.0%) had HT, 11 (4.8%) had DM and HT, and 1 (0.4%) had HL. 

At the end of the second year of follow-up, 22 patients (11.5%) had DM, 20 (10.5%) had HT, 12 (6.3%) had DM and HT, and 1 (0.5%) had HL. In the third year, 12 patients (8.3%) had DM, 9 (6.2%) had HT, 5 (3.4%) had DM and HT, and 1 (0.7%) had HL. In the fourth year, 8 patients (8.4%) had DM, 6 (6.3%) had HT, 5 (5.2%) had DM and HT, and 1 (1.0%) had HL. Finally, in the fifth year, 7 patients (10.9%) had DM, 4 (6.2%) had HT, 4 patients (6.2%) had DM and HT, and 1 (1.5%) had HL.

### 4.8. Metabolic Diseases in Obesity Developed Patients

Forty patients developed obesity after transplantation, of whom 11 (27.5%) had DM, 5 (12.5%) had HT, and 2 (5%) had DM and HT together before transplantation (Figure 2 b). Among the patients who developed obesity after transplantation, 3 (7.5%) developed de-novo DM, 8 (20%) developed HT, and 1 (2.5%) developed HL after transplantation during 5 years of follow-up. Monitoring of patients using TAC and CsA showed no significant difference regarding newly developed diabetes mellitus, hypertension and hyperlipidemia (P = 0.30). Appropriate medical and non-pharmacological (education, diet programs, exercise, etc.) treatment was applied for patients who developed HT, DM, HL, and obesity. During the follow-ups, there were 5 complications: 1 patient had a stroke, 1 patient had diabetic nephropathy and retinopathy and 2 patients had possible hypertensive nephropathy. 

**Table 1.** Mean Body Weight (BW) Before and After Liver Transplantation

	Pre-transplant (n = 226)	Post-transplant						
		1 month (n = 226)	6 months (n = 226)	1 year (n = 226)	2 years (n = 190)	3 years (n = 143)	4 years (n = 95)	5 years (n = 64)
**Body Weight,Mean ±SD(Range)**	72.1±12.9 (40–125)	66.4±11,0 (40–110)	70.6±11.9 (41–110)	73.6±12.4 (42–109)	75.5±12.5 (42–107)	75.7±13.2 (42–107)	74.2±13.9 (45–108)	74.6±14.3 (43–108)
**Male VA, kg,Mean ±SD(Range)**	(n=151) 75.5±11.8 (48-125)	(n=151) 69.0 ± 10.2 (50-110)	(n=151) 73.7 ± 11.3 (48-110)	(n=151) 76.5 ± 11.8 (45-109)	(n=128) 78.0 ± 11.7 (54-107)	(n=94) 78.1 ± 11.9 (49-107)	(n=65) 77.3 ± 12.6 (50-106)	(n=43) 77.2 ± 12.7 (53-106)
**Female Mean ± SD (Range)**	(n = 75) 65.3 ± 12.6 (40–100)	(n = 75) 61.1 ± 10.9 (40–97)	(n = 75) 64.4 ± 10.8 (41–90)	(n = 75) 67.7 ± 11.9 (42–940	(n = 62) 70.3 ± 12.8 (42–106)	(n = 49) 71.0 ± 14.5 (42–105)	(n = 30) 67.7 ± 14.5 (45–108)	(n = 21) 69.0 ± 16.2 (43–108)

**Table 2.** BMI Distribution in Years

	Pre-transplant (n=226)	Post- transplant1 month (n=226)	6 months (n=226)	1 year (n=226)	2 years (n=190)	3 years (n=143)	4 years (n=95)	5 years (n=64)
**BMI, kg/m²,Mean ±SD (Range)**	25.7±4.2 (15.8–41.2)	23.7±3.6 (14.3–37.3)	25.1±3.8 (176.8–37.5)	26.2 ±4.0 (15.2–38.0)	26.7 ±4.2 (15.2–43.0)	26.8 ±4.5 (15.2–41.0)	26.0 ±4.5 (16.4–42.2)	26.4 ±4.8 (15.6–42.2)
**BMI: <20,kg/m², No.(%)**	13 (5.8%)	30 (13.3)	14 (6.2)	10 (14.6)	7 (3.7)	8 (5.6)	7 (7.4)	4 (6.3)
**BMI: 20-24.9,kg/m², No. (%)**	96 (42.5%)	129 (57.0)	108 (47.8)	82 (36.3)	56 (29.5)	38 (26.6)	36 (37.8)	18 (28.1)
**BMI: 25-29.9,kg/m², No. (%)**	84 (37.1)	54 (23.9)	75 (33.2)	97 (42.9)	87 (45.8)	69 (48.2)	34 (35.8)	33 (51.6)
**BMI: ≥30,kg/m², No. (%)**	33 (14.6)	13 (5.8)	29 (12.8)	37 (16.4)	40 (21.0)	28 (19.6)	18 (19.0)	9 (14.0)
**BMI, kg/m², Mean Male (n=151)**	25.6	23.4	25.0	26.0	26.3	26.2	25.9	26.1
**BMI, kg/m², Mean Female (n = 75)**	25.7	24.1	25.4	26.7	27.7	27.9	26.3	(n=43)27.0 (n=21)

**Table 3.** Known and New Obese Cases in Post-Transplantation Follow-Up

	Preop^a^ (n=226)	Postop^a^ (n=226)	6m^a^ (n=226)	12m^a^ (n=226)	24m^a^ (n=190)	36m^a^ (n=143)	48m^a^ (n=95)	60m^a^ (n=64)
**Obese-remain obese patients, %**	14,60	5,75	5,31	4,42	5,26	4,90	3,16	4,69
**Total obese patients (known and new cases), %**	14,60	5,75	12,83	16,37	21,05	19,58	18,95	14,06

^a^ Abbreviations: Preop, before liver transplantation; Postop, after liver transplantation; m, month

**Table 4.** Factors Associated With Obesity After Liver Transplantation in Multivariate Analysis

	B	SE	P value	OR	95.0% CI	
					Lower	Upper
**When pre-transplantation obese group was included**						
Pre-Ltx^a^ obese patients	1,48	0,32	0,000	4,37	2,33	8,19
Age	0,04	0,02	0,042	1,04	1,00	1,08
Tacrolimus	-0,07	0,01	0,000	0,94	0,92	0,96
Cyclosporine	-0,05	0,01	0,000	0,95	0,93	0,97
**When pre-transplantation obese group was excluded**						
Sirolimus	-0,05	0,01	0,000	0,95	0,93	0,97
Age	0,09	0,03	0,002	1,09	1,03	1,16
Gender	1,04	0,43	0,017	2,83	1,21	6,61
Tacrolimus	-0,08	0,02	0,000	0,92	0,89	0,95
Cyclosporine	-0,07	0,02	0,000	0,93	0,90	0,97
Sirolimus	-0,06	0,02	0,000	0,94	0,91	0,97

^a^ Abbreviation: Pre-Ltx, Before liver transplantation

**Table 5.** Obesity Development in Patients Using CNI

	TAC^a^, No. (%):	CsA^b^, No. (%):	Total, No. (%)	P value
**Pre-transplant non-obese patients (BMI<30 kg/m²)**	117(79)	31(21)	148(100)	
**Post-transplant patients developing obesity**	21(17.9)^c^	6 (19.4)^c^	27(18.2)	0.07^c^

^a^ The patients who took only TAC-based therapy from CNI during their follow-up period (not changed to CsA during treatment)

^b^ The patients who took only CsA-based therapy (not changed to TAC during treatment)

^c^ TAC vs CsA; P: 0.07

## 5. Discussion

In this study we showed that the number of patients who had obesity increased by up to 21% in the second year after LTx. The prevalence of obesity decreased to pre-transplantation rates during the fifth year. Pre-transplant obesity prevalence was 14.6% and post-transplant prevalence at the 5th year was 4.9%. Patient’s education and medical nutrition therapy may be associated with the improvement. Obesity prevalence at the end of the 5 year follow up was found to be 14.06% with the addition of new subjects after liver transplantation (Table 3, Figure 1).

Owing to improvements in surgical techniques, post-operative care and new immunosuppressive treatments, liver transplantation has become the most effective method for acute or chronic end-stage liver disease patients. Since liver transplantation extends the survival of most patients, it necessitates long-term follow-up and care ( 1 ). As survival after liver transplantation gets longer, weight gain and obesity have become common health problems for organ recipients. Obesity is seen in approximately 20% of patients after liver transplantation ( 6 ) compared to 20–28% in the general population (9 , 10). Although immunosuppressive medications used after liver transplantation have many known side effects, immunosuppressives’ effect on weight gain has not yet been clarified (8). Some researchers state that there is no significant difference, however there is also contrary literature stating otherwise (4 ,11 ,12). We utilized weight gain and BMI measurements similar to studies in the literature that evaluated weight gain after transplantation. We did not use waist and hip circumference measurements due to the fact that, in relation to the severity of pre-transplantation diseases, there might be ascites in the abdominal cavity as a result of cirrhosis. Retrospective data of 226 patients in our study showed an average decrease of 5.7 ± 6.2 kg in the 1st month of post-operative control when compared to the pre-operative period. This situation can be explained by ascites and pretibial edema in patients before liver transplantation. Since patients’ mean dry weights were not measured, we can also speak of a relative weight loss due to drainage of ascites from the peritoneal cavity after the operation. In addition to this, pre-operative time spent in intensive care units, emotional status of the patients, and insufficient nourishment caused by medications may be important factors in post-operative weight loss. The patients who used a steroid for six months showed a mean weight gain of 4.71 ± 5.75 kg vs. 2.77 ± 6.98 kg for the non-steroid group of patients. The difference between these 2 groups was statistically significant (P = 0.03). In a prospective study by Richards et al., in which 597 liver transplantation patients were evaluated for weight change, a rapid weight gain was reported in the first year, 5 kg in the second year, and 10 kg in the third year ( 4 ). The first month’s BMI measurement of our patients after liver transplantation was 23.7 ± 3.6 kg/m², significantly increasing to 26.2 ± 4.0 kg/m² in the following year. 17.6% of patients included in the study developed obesity 2 years after transplantation. Likewise, 21.6% of the non-obese patients in the study conducted by Everhart et al. developed obesity 2 years after transplantation ( 11 ). Thus, our findings were similar to the studies in the literature ( 6 , 11 ). Various studies state that obesity is seen less often in patients using tacrolimus than in patients using cyclosporine after liver transplantation ( 13 ). Although some studies relate CsA use directly to obesity development ( 4 , 14 ), other studies do not accept this theory ( 15 , 16 ). In a study by Canzanello et al, 46% of patients using CsA developed obesity compared to 27% in TAC users ( 17 ). The fact that more CsA users developed obesity than TAC users was probably because TAC users needed corticosteroids more ( 7 ). Most of the patients in our study used TAC-based (73.8%) or CsA-based (24.7%) immunosuppressive treatments. The prevalence of MMF and steroid treatments in addition to calcineurin inhibitors was 88.0% and 95.5%, respectively. Among patients who were not obese (BMI < 30 kg/m²) at the time of liver transplantation and used CNI, 18.2% developed obesity. In contrary to the literature, no statistically significant difference in mean weight gain was found between TAC users and CsA users ( 12 ). There was also no significant difference in development of obesity between the patients using tacrolimus and cyclosporine (17.9% vs. 19.4%, P = 0.07). Multivariate analysis showed that TAC, sirolimus and CsA were negatively associated with the development of obesity (P < 0.001, P < 0.001 and P < 0.001). 

In our study we showed that age and gender were independent variables that predicted the development of obesity. (P = 0.002 and P = 0.013). Subjects with pre-transplantation obesity had 4.37 times higher risk of post-transplantation obesity. Therefore subjects with pre-transplantation obesity should be strictly followed. Regular evaluation of metabolic parameters and initiation of medical nutrition therapy can be beneficial in the long term. In multivariate analysis, when the pre-transplantation obese group was excluded, it was demonstrated that age, gender, Tac, CsA ve sirolimus significantly predicted obesity. In this group of subjects, risk of developing obesity was found to be 2.87 times higher in men than in women. Remaining factors had weak effects on obesity. Therefore, male subjects should be evaluated strictly in terms of obesity after transplantation. Although some researchers state that immunosuppressives, especially corticosteroids, are responsible for weight gain and obesity development after transplantation (11, 17), other researchers do not support this view (4, 18). The common opinion is that the appetizing effect of steroids contributes to weight gain (19). The study of Wawrzynowicz et al. on 75 patients who had liver transplantation showed weight gain of 6.1 kg in the first 6 months after transplantation and a dynamic weight gain occurred in the first year (8). With regards to weight gain, there was no significant difference between steroid users and non-users. Furthermore, the same study did not identify a significant weight gain difference between use of cyclosporine and tacrolimus. Contrary to the study of Wawrzynowicz et al., our study identified an average increase of 4.71 kg in the body weight of the group who used a steroid for 6 months and 2.77 kg in the group who did not use a steroid (P = 0.03). Furthermore, a significant increase in the body weight has persisted between the two groups in the first year after liver transplantation (P = 0.02). On the other hand, some studies state that weight gain continues even though steroid use is decreased (18). The retrospective data of our patients showed that among the patients who developed obesity after transplantation, 3 (7.5%) developed de-novo DM, 8 (20%) developed HT, and 1 (2.5%) developed HL after transplantation. Although there are publications stating that tacrolimus is more diabetogenic than cyclosporine (20), our study did not find a significant difference regarding newly developed diabetes mellitus, hypertension and hyperlipidemia due to TAC and CsA use (P = 0.30). The study by Sheiner et al. on 139 liver transplantation patients did not observe an effect of CNIs on new diabetes development; the same publication found that the rate of weight gain and hypercholesterolemia due to immunosuppressive treatment was similar to that of the general population (12). The study by Becker et al. which compared tacrolimus/daclizumab and tacrolimus/MMF immunosuppressive treatments stated that not using a steroid decreased de-novo diabetes development and insulin use after transplantation and the patients did not gain weight (21). Another factor in obesity development after transplantation is the changes in nourishment. Termination of the limited dietary program before transplantation, the patient’s emotional status, and the appetizing effect of corticosteroids contribute to this. The effect of MMF treatment on obesity was not suitable for statistical evaluation, as most of the patients (88%) received this treatment (in addition to CNI treatment) and there were only a few patients whom we could exclude. There are some limitations in our study; the patient population was relatively small, our study was retrospective in nature and all of the patients were not followed up throughout the five years. In conclusion, our study showed that the prevalence of obesity in patients with liver transplantation is 21% and 14% at the end of the second year and the fifth year, respectively. Non-corticoid immunosuppressive medications (CNI, mTOR inhibitors, MMF) did not have a significant effect on weight gain and obesity development. The reason why there was no significant difference between TAC and CsA with regards to weight gain may be explained by the fact that our patients who used CNI in the first years after liver transplantation received steroid treatment as well. Along with corticosteroid treatments in the first months, we think that a sedentary lifestyle, recovering hepatic function, regained appetite, and improved nourishment also play a role in weight gain and obesity.
